# Elastic Response of Open-Hole Material-Extruded PLA Plates: Equivalent Laminate Architectures and Perimeter Paths

**DOI:** 10.3390/polym18182301

**Published:** 2026-09-20

**Authors:** Mehmet Emin Baysal, Yasin Uslugil

**Affiliations:** 1 Department of Industrial Engineering, Konya Technical University, 42250 Konya, Türkiye; 2 Department of Mechatronics Engineering, KTO Karatay University, 42020 Konya, Türkiye; yasin.uslugil@karatay.edu.tr

**Keywords:** additive manufacturing, material extrusion, polylactic acid, mechanical properties, finite element analysis, stress concentration, laminate architecture

## Abstract

The elastic response of an open-hole material-extruded plate depends on both its interior raster architecture and the deposition paths around its boundaries. This study examines whether laminate preferences obtained from a homogeneous model persist when perimeter paths are represented. Six symmetric equivalent-laminate architectures were compared for polylactic acid plates at hole-diameter-to-width ratios of 0.20, 0.25, and 0.40 using plane-stress finite-element models with homogeneous interiors, tangential hole bands, and combined hole and outer bands. Mesh refinement, an independent block mesh, and layered-shell comparisons assessed numerical consistency. In the finest homogeneous comparison at a ratio of 0.25, [90/45/90] reduced the stress concentration factor by 0.90% relative to [0/0/0], while increasing displacement by approximately 2.84%. Adding hole and outer bands reversed this stress advantage, giving a 1.38% higher stress concentration for [90/45/90]. Across the complete perimeter-model screen, [0/0/0] gave both the lowest stress concentration and the lowest displacement, eliminating the preference-dependent trade-off found in the homogeneous models. Comparison with published tensile curves gave elastic-slope discrepancies of 0.5–3.9% for the primary material dataset and 13.2–16.0% for the experimental source’s constants; this literature-based benchmark is limited to global elastic response and does not validate local hole-edge stresses, failure loads, or laminate ranking. Perimeter paths can therefore determine the preferred elastic architecture even when the interior material exhibits only weak orthotropy.

## 1. Introduction

Material extrusion (MEX), including filament-based fused deposition modeling and fused filament fabrication, builds components from oriented roads and stacked layers. For polylactic acid (PLA), road alignment, inter-road bonding, air gap, and deposition conditions affect the effective stiffness tensor. Experimental measurements identify orthotropy even when air gap control reduces voids, and show that Poisson ratios depend on print direction and speed [1,2]. An elastic description must therefore retain the material directions and the print conditions associated with its constitutive data.

Open-hole response involves both a geometric stress concentration and a deposition architecture. Experiments and simulations on one- and two-hole PLA plates have examined local stress, hole size, and structural strength [3,4,5,6]. Framed thermoplastic specimens show different strength and elongation responses for axial and diagonal rasters [7]. Printed and drilled holes also differ in tensile resistance because their local paths and discontinuities differ [8]. Elastic–plastic comparisons on PLA+ further demonstrate the importance of matching the material formulation and constitutive regime [9]. These results motivate explicit treatment of perimeter direction when comparing otherwise similar elastic architectures.

Published tests on orthotropic PLA with printed and drilled holes provide a basis for an external response comparison [10]. That study used linear-elastic stresses together with a stress-gradient criterion to predict failure. An elastic tensile slope, a local stress concentration factor, and a failure load test different aspects of a model. Accordingly, experimental stiffness agreement must be distinguished from validation of a failure mechanism or of a laminate ranking.

Microstructural homogenization transfers the effective response of deposited roads to a structural model [11]. Classical laminate theory provides a related thickness reduction for polymer parts with different raster orientations [12,13,14]. Temperature and layer geometry affect road cohesion [15,16], while a hole alters the load redistribution among orientation families [17]. Curved deposition trajectories have improved open-hole performance in reinforced thermoplastics [18,19,20]. Those systems do not supply transferable PLA strengths, but establish why local path direction merits a separate comparison with uniform material axis models.

Architecture selection also requires a trade-off between stress and deformation. Multi-attribute methods in additive manufacturing organize a finite set of alternatives and explicit preferences [21,22]; feasibility filtering prevents compensation by an attractive but inadmissible attribute [23]. Here, the stress concentration factor $K_{t}$ is the maximum longitudinal stress near the hole divided by gross-section nominal stress, and $u_{\max}$ is the maximum in-plane displacement magnitude. The geometry parameter D/W denotes hole diameter divided by plate width. These elastic quantities define a response-constrained comparison, without substituting for strength or manufacturing cost data.

The present study examines six equivalent-laminate architectures at three hole ratios, with homogeneous, hole–perimeter, and combined hole-and-outer-perimeter representations. Mesh and formulation checks assess whether the small paired differences can be resolved numerically; published tensile curves assess the global elastic response. Tensile–compressive asymmetry, interlayer fracture, and raster-dependent toughness remain distinct constitutive mechanisms [24,25,26,27]. The resulting architecture preferences are resolved for the specified elastic properties, deposition paths, and permitted displacement.

Constitutive modeling of material-extruded parts has consequently progressed from idealized orthotropy toward representations that retain build strategy, local material axes, and process-dependent architecture [28,29,30]. This distinction is relevant here because a homogeneous laminate reduction and a perimeter-aware representation answer different structural questions. At the interlayer scale, auxiliary heat alters the bonding and mechanical response of FDM polylactide [31], while PLA failure modeling has also required a separate strength and failure-mode description beyond elastic constants [32]. These studies support separating the present elastic comparison from claims about fracture resistance.

The broader additive manufacturing literature likewise shows that interface formation can be a governing design variable. Ganguly and Tang demonstrated that solvent-exchange postprocessing can substantially alter the performance of a printed nanocomposite [33]; Wu and Song reviewed how solid–liquid interfacial interactions govern process optimization in DLP printing [34]. Together, these studies establish interface formation as a process-specific design variable. They therefore frame the perimeter and interfacial idealizations used here, while the constitutive comparison remains specific to the MEX PLA open-hole configuration.

## 2. Materials and Methods

### 2.1. Geometry, Loading, and Response Measures

The plate length *L*, width *W*, and thickness *t* were 100, 40, and 3 mm. Diameters D=8, 10, and 16 mm give D/W=0.20, 0.25, and 0.40, respectively; 10 mm is the baseline diameter. The global *x* axis is the loading direction and the origin is the hole centre. The left edge had zero *x* displacement, and its node nearest the transverse midpoint had zero *y* displacement to remove rigid translation. The right edge carried uniform nominal traction $\sigma_{\mathrm{nom}}=10\mathrm{MPa}$. Geometry and loading remained fixed when material axes changed. The calculations used mm, N, and MPa.

The response definitions were $K_{t}=\max_{r\leq R}\left(\sigma_{x}\right)/\sigma_{\mathrm{nom}}$ and $u_{\max}=\max\sqrt{u_{x}^{2}+u_{y}^{2}}$, where $r=\sqrt{x^{2}+y^{2}}$, *R* is the search radius, $\sigma_{x}$ is longitudinal stress, and $u_{x},u_{y}$ are displacement components. The default R=15mm contains all hole boundaries, whose radii range from 4 to 8 mm, and excludes the loaded and supported ends at |x|=50mm. Radii of 9, 12, 15, and 18 mm were compared. Gross-section stress was checked independently as the total axial reaction magnitude divided by $Wt=120{\mathrm{mm}}^{2}$; the expected reaction magnitude is 1200 N.

### 2.2. In-Plane Orthotropic Constitutive Model

The plane-stress material model was defined by $E_{1}$, $E_{2}$, $G_{12}$, and $\nu_{12}$, then rotated into the plate coordinates for each material axis angle. Table 1 gives the zero-air-gap values reported in Table 4 of Li et al. [1].

Reciprocal symmetry yields $\nu_{21}=\nu_{12}E_{2}/E_{1}$, and the in-plane constitutive relation becomes

(1) $${\left[\begin{array}{ccc} \sigma_{1} & \sigma_{2} & \tau_{12} \end{array}\right]}^{T}=\left[\begin{array}{ccc} Q_{11} & Q_{12} & 0\\ Q_{21} & Q_{22} & 0\\ 0 & 0 & Q_{66} \end{array}\right]{\left[\begin{array}{ccc} \varepsilon_{1} & \varepsilon_{2} & \gamma_{12} \end{array}\right]}^{T},\;\begin{array}{cccc} Q_{11} & =E_{1}/\Delta , & Q_{22} & =E_{2}/\Delta ,\\ Q_{12} & =\nu_{12}E_{2}/\Delta , & Q_{66} & =G_{12}, \end{array}$$

where $E_{1}$ and $E_{2}$ are the longitudinal and transverse Young moduli, $G_{12}$ is the in-plane shear modulus, and $\nu_{12}$ and $\nu_{21}$ are reciprocal Poisson ratios. The normal stresses and strains are $\sigma_{j}$ and $\varepsilon_{j}$ (j=1,2); $\tau_{12}$ and $\gamma_{12}$ denote shear stress and engineering shear strain. The reduced stiffness coefficients are $Q_{ij}$ and $\Delta =1-\nu_{12}\nu_{21}$. Elastic reciprocity requires $Q_{21}=Q_{12}\neq 0$; only the local normal–shear coupling terms vanish. For the selected material, $\nu_{21}=0.416$, Δ=0.821, and $\left(Q_{11},Q_{22},Q_{12},Q_{66}\right)=\left(3250.7,3145.9,1352.8,919.0\right)$ MPa. The transformed stiffness $\bar{Q}\left(\theta \right)$ was calculated using the standard stress and engineering-strain transformation matrices and supplied to the element formulation. Thus, material axis rotation changes the constitutive tensor while preserving the geometry, mesh, boundary conditions, and global loading frame. Angles refer to material axes measured from the loading direction, not to rotation of the plate. Zhao et al. independently reported that the elastic modulus of MEX PLA changes with printing angle and layer thickness, which reinforces the need to retain print-condition-specific material inputs [35].

### 2.3. Three-Ply Laminate Reduction

The thickness was partitioned into three equal equivalent plies, each representing a group of deposited layers with a common orientation. The following three-ply candidates were restricted to symmetric sequences, so that the membrane–bending coupling matrix vanishes and a plane-stress comparison is physically consistent: [0/0/0], [0/90/0], [45/−45/45], [0/45/0], [90/45/90], and [45/0/45]. For an individual ply, the transformed reduced stiffness is ${\bar{Q}}^{\left(k\right)}\left(\theta_{k}\right)$. The laminate extensional stiffness and thickness-normalized equivalent stiffness are

(2) $$A=\sum_{k=1}^{N}{\bar{Q}}^{\left(k\right)}\left(z_{k}-z_{k-1}\right),\;Q^{\mathrm{eq}}=\frac{A}{h},\;h=\sum_{k=1}^{N}\left(z_{k}-z_{k-1}\right).$$

Here, *N* is the number of equivalent plies, $z_{k}$ and $z_{k-1}$ are their upper and lower thickness coordinates measured from the laminate mid-plane, *h* is total thickness, A is extensional stiffness, and $\theta_{k}$ is the orientation of ply *k*. With equal 1 mm plies, $Q^{\mathrm{eq}}$ is the arithmetic mean of the three transformed ply stiffness matrices. The reduction preserves the orientation-fraction-dependent in-plane stiffness and the ${\bar{Q}}_{16}$ and ${\bar{Q}}_{26}$ coupling terms, but omits bending, delamination, and ply failure. Prior MEX laminate implementations have used the same CLT basis [12,13,14]. Symmetry removes the membrane–bending coupling matrix B, but does not require $A_{16}$ or $A_{26}$ to vanish. Permuting plies while retaining their orientation fractions leaves A/h unchanged; the membrane model therefore does not resolve stacking-order effects at fixed fractions. Table 2 lists the in-plane constants supplied to MAPDL.

### 2.4. Deposition Paths and Physical Layer Interpretation

Table 3 defines every architecture code. The three 1 mm equivalent plies describe orientation fractions, not three physical extrusion layers. Deposited-layer height $h_{\ell }$, nozzle diameter, road width, and in-plane element size are distinct quantities. At an illustrative $h_{\ell }=0.2\mathrm{mm}$, a 3 mm plate contains 15 deposited layers and each equivalent ply represents five such layers. A 0.4 mm nozzle diameter alone does not determine this count. Subdividing each equivalent ply into five or ten identical-orientation subplies gives 15- and 30-layer shell representations at unchanged total thickness and orientation fractions.

**Figure 1:**
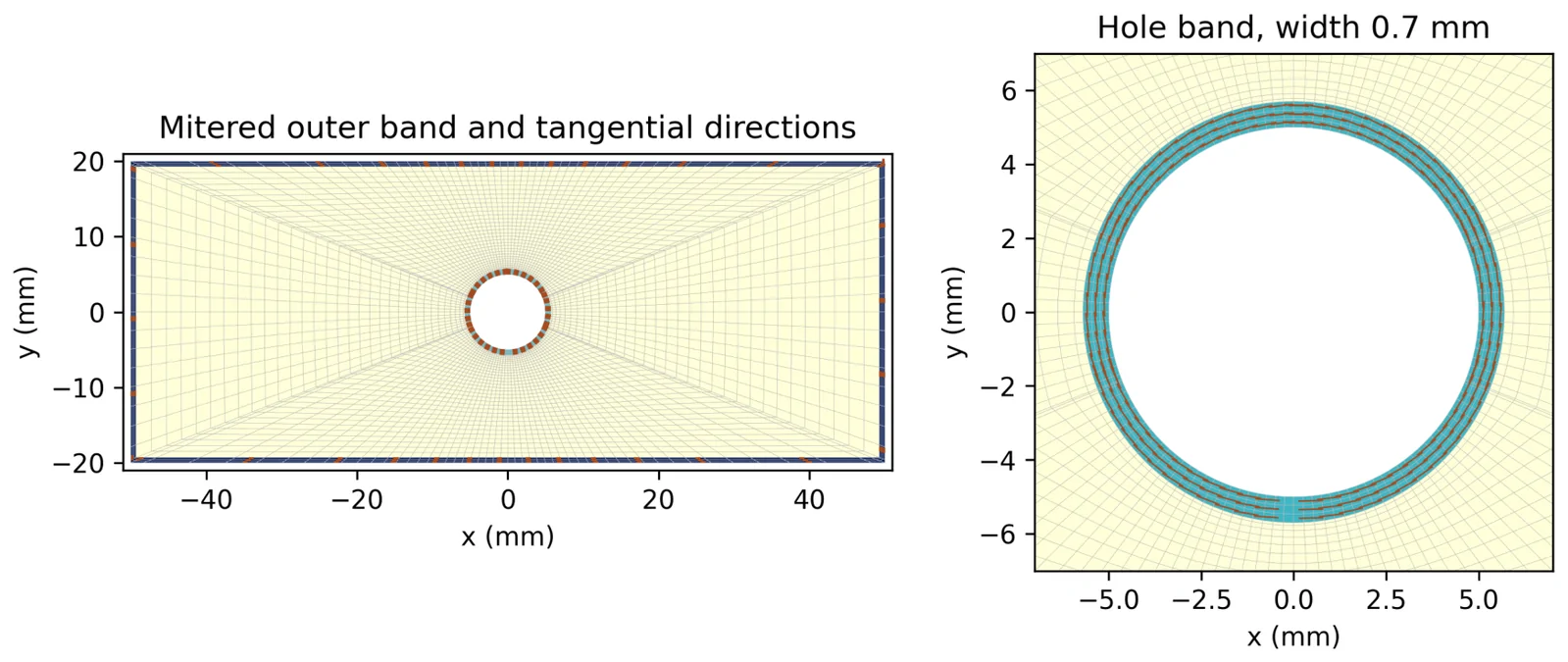
Perimeter regions and material directions in the PHO model.

The perimeter scenarios retain the same geometry and thickness. Within a hole band, the local material axis follows the circular tangent; the outer band follows the nearest straight edge with mitered transitions at corners. All equivalent plies use this local tangent within the band, while the interior retains the candidate stiffness. Interfaces are perfectly bonded and void-free. Band widths of 0.7 and 1.4 mm represent two or four adjacent roads of 0.35 mm width. The road width and illustrative 0.2 mm layer height are taken from the experimental processing description in [10]; the perimeter counts define controlled path scenarios. They isolate the structural consequence of local directions while holding the geometry, thickness, and elastic inputs fixed. Figure 1 shows the PHO regions and material directions, with the near-hole view resolving the 0.7 mm tangential band.

### 2.5. Finite-Element Discretization and Verification

**Figure 2:**
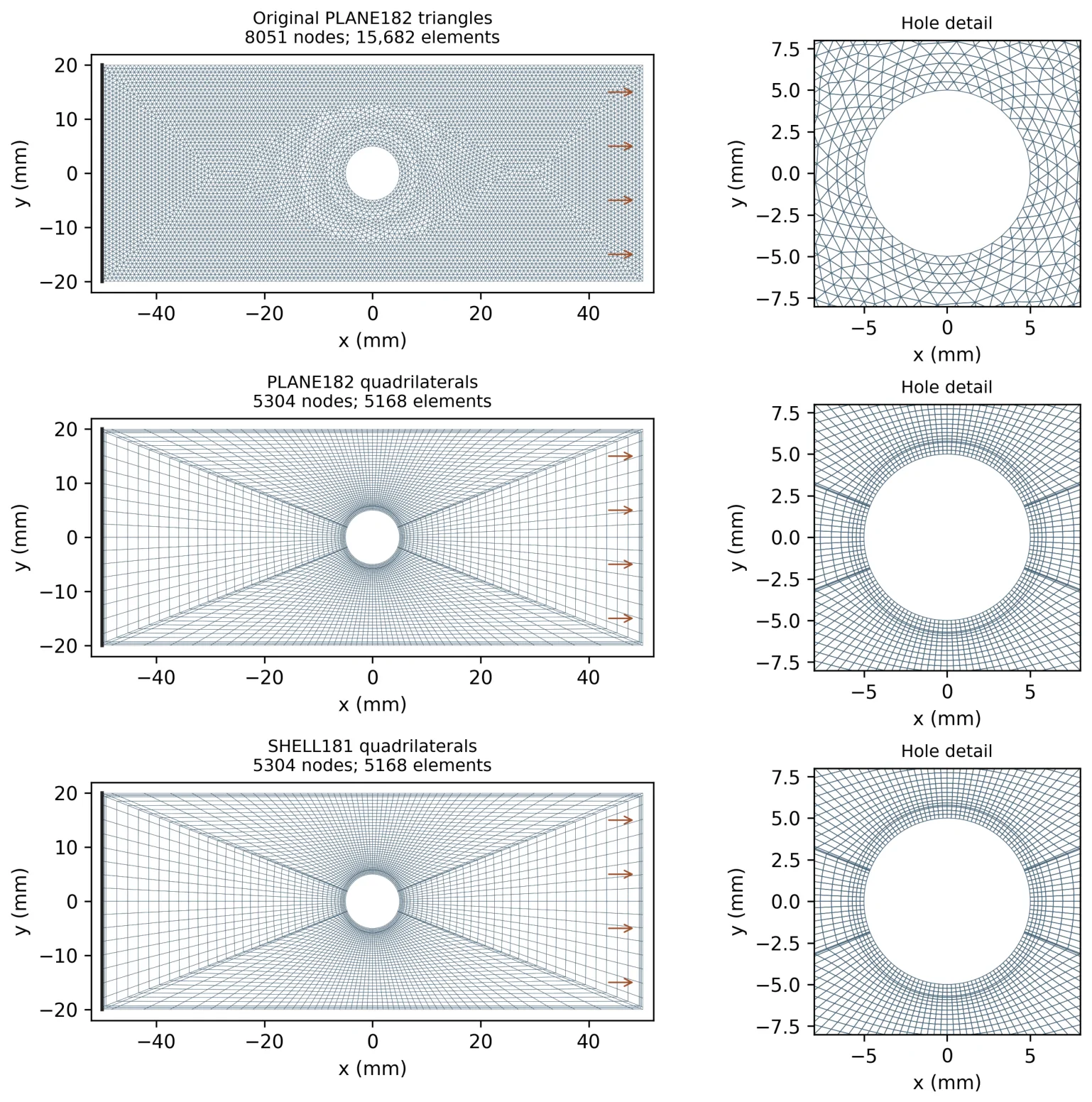
Representative full-domain and near-hole meshes for PLANE182 and SHELL181. Orange arrows indicate the applied uniform nominal traction on the right edge.

ANSYS MAPDL 2026 R1 was automated using PyMAPDL 0.74.0 with Python 3.11. PLANE182 is a four-node structural solid element used here in two-dimensional plane stress with translational degrees of freedom. Explicit thickness was activated using KEYOPT(3) = 3 and t=3mm. An initial free mesh used degenerate triangular PLANE182 elements. The revised comparison used mapped quadrilaterals, with circumferential divisions $n_{\theta }=128$, 256, 512, and, for the three core architectures at the baseline diameter, 1024. The complete six-candidate, three-diameter, three-perimeter screen used full-integration PLANE182 with KEYOPT(1) = 0 at $n_{\theta }=512$. The finer core comparison used enhanced-strain KEYOPT(1) = 2. Comparisons are made within the same formulation and resolution. Figure 2 shows representative full-domain and near-hole views of the initial triangular PLANE182, mapped quadrilateral PLANE182, and layered SHELL181 meshes; the displayed meshes are not the finest resolution.

**Figure 3:**
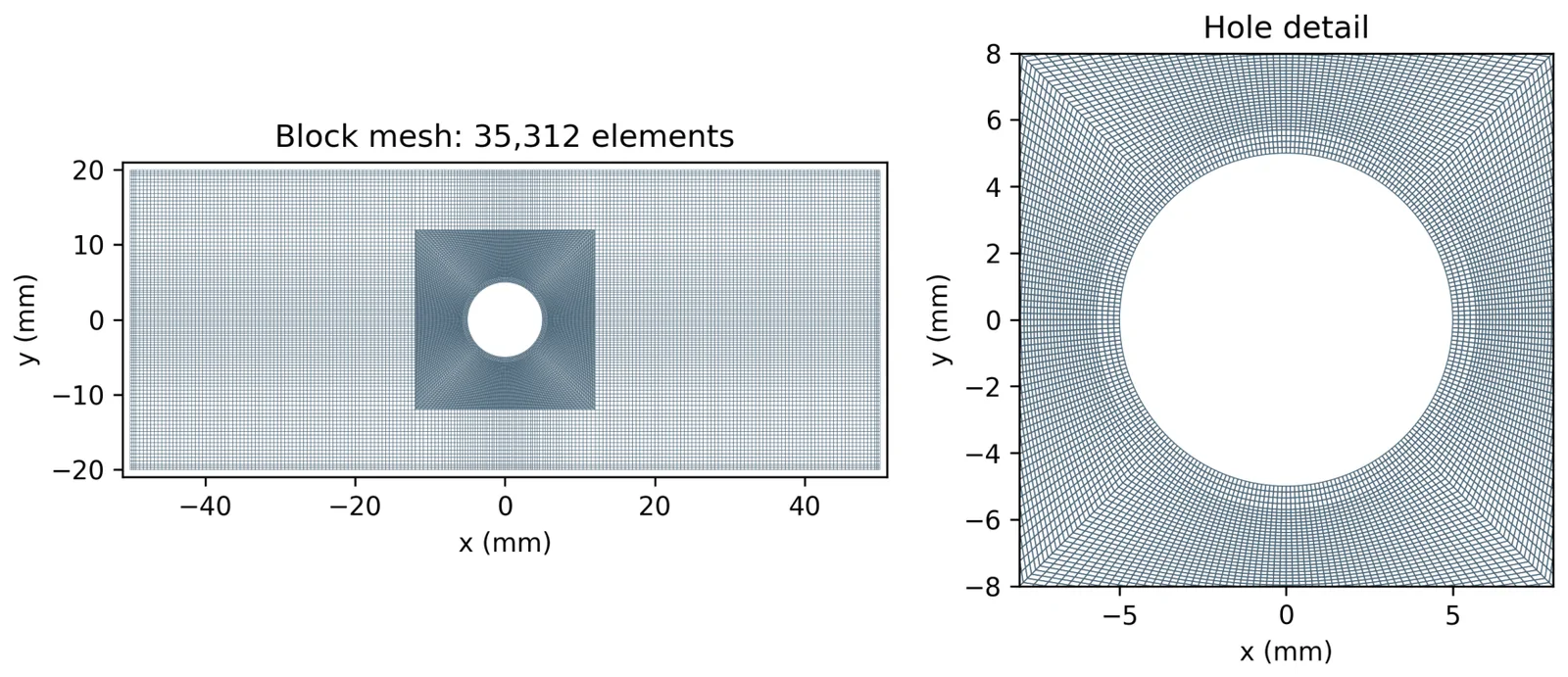
Independent block mesh for the topology sensitivity check.

The finest core mesh contained 298,248 nodes and 297,216 elements. An independent mesh joined a circular inner region to a square at x,y=±12mm and Cartesian outer blocks. The independent topology shown in Figure 3 tests the effect of narrow radial elements near outer corners in the original mapped mesh. The independent $n_{\theta }=512$ mesh contained 139,592 nodes and 138,704 elements. Nodal averaged and element-nodal unaveraged longitudinal stresses, reaction equilibrium, and peak position were checked. Perimeter-angle increments of 2.5, 1.25, and 0.625 degrees were also compared at fixed mesh resolution.

SHELL181 is a four-node layered structural shell with translational and rotational degrees of freedom. Three core P0 architectures were checked using full-integration shells, KEYOPT(3) = 2, with explicitly fixed global material reference axes and the prescribed ply rotations. The shell and enhanced-strain plane models used matched quadrilateral meshes, thickness, loading, and in-plane constraints. Shell edge load was $\sigma_{\mathrm{nom}}t=30\;N/\mathrm{mm}$. The left shell edge had zero out-of-plane displacement, and rotations at the anchor node were constrained to remove rigid-body modes. The auxiliary out-of-plane constants were $E_{3}=1000$ MPa, $G_{13}=G_{23}=500$ MPa, and $\nu_{13}=\nu_{23}=0$; they are modeling inputs for this membrane check, not experimentally identified through-thickness properties. Longitudinal stresses evaluated at the mid-surface of each equal-thickness ply were averaged through thickness before extracting $K_{t}$. For either response $y\in \{K_{t},u_{\max}\}$, the percentage discrepancy is

(3) $$\delta_{y}=100\frac{y_{\mathrm{shell}}-y_{\mathrm{plane}}}{y_{\mathrm{plane}}}.$$

The 3-, 15-, and 30-layer comparisons preserve orientation fractions and total thickness. They test membrane equivalence under perfect bonding while holding process-induced bond quality outside the comparison.

### 2.6. Published Experimental Benchmark

The drilled, $0^{\circ }$ PLA specimen with a 6 mm hole in Schmeier et al. [10] was selected for the elastic comparison. Its width and thickness are 24 and 2 mm; the gauge length is 100 mm, within a 140 mm specimen. Reported processing includes an Ultimaker S5, nozzle temperature 185 °C, bed temperature 55 °C, speed 45 mm/s, 0.2 mm layer height, 0.35 mm road width, and 100% infill. The benchmark uses the drilled hole geometry without an assumed printed hole perimeter.

Two separately visible traces in Figure 13b of that study [10] were digitized over the preselected strain interval 0.002–0.006. The third overlapping trace could not be recovered independently. The original image axes were mapped to strain and gross-section nominal stress; a two-pixel reading range corresponds approximately to 0.259 MPa and 0.000075 strain. Straight-line fits included an intercept. Experimental apparent stiffness is the fitted slope $E_{\mathrm{app},\exp}$, while the finite-element stiffness is $E_{\mathrm{app},\mathrm{FE}}=\sigma_{\mathrm{nom}}/\left(\Delta L/L_{g}\right)$, where ΔL is end extension and $L_{g}=100\mathrm{mm}$. The comparison interprets the plotted strain as this gauge-average strain; the source does not specify the hole-test strain measurement sufficiently to establish an exact instrument-to-model match.

Both the primary Li constants in Table 1 and the experimental study’s constants ($E_{1}=2330\mathrm{MPa}$, $E_{2}=2140\mathrm{MPa}$, $G_{12}=1040\mathrm{MPa}$, $\nu_{12}=0.375$) were evaluated without fitting either dataset to the hole curves. Transverse end motion was either free apart from a rigid-body constraint, or suppressed at the ends. Refinement used PLANE182 and quadratic PLANE183, with the latter refined from 0.25 to 0.125 mm. The signed slope error is $100(E_{\mathrm{app},\mathrm{FE}}/E_{\mathrm{app},\exp}-1)$. Pointwise mean absolute percentage error (MAPE) is the mean of $100|\sigma_{\mathrm{FE},j}-\sigma_{\exp,j}\left|/\right|\sigma_{\exp,j}|$ at the digitized strains *j*. Slope and pointwise errors are reported separately because an intercept affects the latter.

### 2.7. Property Sensitivity and Response-Constrained Selection

A preceding 100-set Latin-hypercube study varied $E_{1},E_{2},G_{12}$ independently by ±10% and $\nu_{12}$ by ±5% around the primary dataset. Its 300 unique solutions compare UD0, Angle, and H90-45-90 on the common 0.75 mm triangular mesh at D/W=0.25. These retained calculations are a coarse-mesh property sensitivity study, not a finely resolved manufacturing uncertainty distribution. The prescribed intervals are numerical perturbations without measured probabilities.

For each geometry and perimeter scenario *g*, the candidate set is $A_{g}$, and $u_{0,g}$ denotes its UD0 displacement. A permitted displacement increase *c*, expressed in percent, defines the feasible set

(4) $$F_{g}\left(c\right)=\left\{i\in A_{g}:100\left(u_{i}-u_{0,g}\right)/u_{0,g}\leq c\right\}.$$

Candidate *i* has responses $K_{t,i}$ and $u_{i}$. The selected feasible candidate minimizes

(5) $$S_{i}=w{\hat{K}}_{i}+\left(1-w\right){\hat{u}}_{i},\;0\leq w\leq 1,$$

where *w* is the prescribed stress-priority weight. For either response *y*, ${\hat{y}}_{i}=\left(y_{i}-y_{\min}\right)/\left(y_{\max}-y_{\min}\right)$, with extrema taken only over $F_{g}\left(c\right)$; a zero response span contributes zero. Ties are resolved by lower $K_{t}$, then lower displacement. This combines feasibility filtering with explicit multi-attribute preferences [22,23]. The revised decision maps use all six candidates at each diameter and perimeter scenario, with c=0–3.5% in 0.05% increments and w=0–1 in 0.01 increments. The previous coarse-mesh continuous-angle recommendations are excluded from the refined angle assessment.

## 3. Results and Discussion

### 3.1. Numerical Resolution and Membrane Equivalence

Refinement changed the absolute stress concentration appreciably relative to the initial triangular model. For baseline UD0, the initial 0.75 mm mesh gave $K_{t}=3.0275$, whereas the finest enhanced-strain quadrilateral model gave 3.2732. The near-three isotropic result is therefore insufficient as a convergence criterion for this finite-width plate. The refined values provide the absolute stress and architecture comparisons reported here.

Between $n_{\theta }=512$ and 1024, core-model stress concentrations changed by approximately 0.15%. More relevant to ranking, the changes in paired percentage contrasts were at most 0.0032 percentage points. The independent block mesh agreed with the corresponding enhanced-strain mapped mesh within 0.003% at $n_{\theta }=512$, and all 12 block-mesh checks completed without element-shape warnings. These comparisons support the reported paired ordering within each model, without assigning a formal confidence interval to discretization error.

Search radii of 9, 12, 15, and 18 mm returned the same peak $K_{t}$ in the completed comparisons; maxima remained on the hole boundary. The nominal stress reconstructed from reactions differed from 10 MPa by less than $1.3\times 10^{-9}$ MPa. Explicit plane-stress thickness changed the reaction from the unit-thickness value to 1200 N, while leaving traction-controlled stress and displacement unchanged. Angle quantization in the perimeter bands changed $K_{t}$ by approximately 0.002% over the tested increments.

Table 4 compares matched membrane formulations at $n_{\theta }=512$. Across the tested resolutions, shell-to-plane differences remained below 0.030% for $K_{t}$ and 0.0011% for displacement. Explicitly fixing the shell material axes removed a dependence on element-local orientation. Subdivision into 15 or 30 layers produced the same membrane responses to numerical precision, as expected from unchanged A/h. This equivalence applies to the membrane representation; physical layer height remains a process variable governing printed bond properties.

### 3.2. Architecture and Perimeter Interaction

Table 5 gives the complete baseline six-candidate comparison with the same full-integration formulation and mesh for every entry. Without perimeters, H90-45-90 has the lowest $K_{t}$ and UD0 the lowest displacement. The hole-and-outer-perimeter representation reverses the stress ordering: UD0 has the lowest $K_{t}$ and H90-45-90 the highest. Thus, the choice of local deposition direction changes the conclusion even though the far-field material constants and geometry are unchanged.

Table 6 reports the enhanced-strain core results at the finest resolution. In the finest core P0 comparison, Angle and H90-45-90 reduce $K_{t}$ relative to UD0 by 0.680% and 0.899%, respectively. Their displacements increase from the UD0 value of approximately 0.39965 mm to 0.40738 and 0.41101 mm. For PHO, Angle and H90-45-90 instead exceed UD0 stress concentration by 0.895% and 1.376%. Adding the bands changes H90-45-90 from $K_{t}=3.24372$ to 3.31089, a 2.071% increase. The perimeter effect therefore exceeds and reverses the small homogeneous-model advantage.

**Figure 4:**
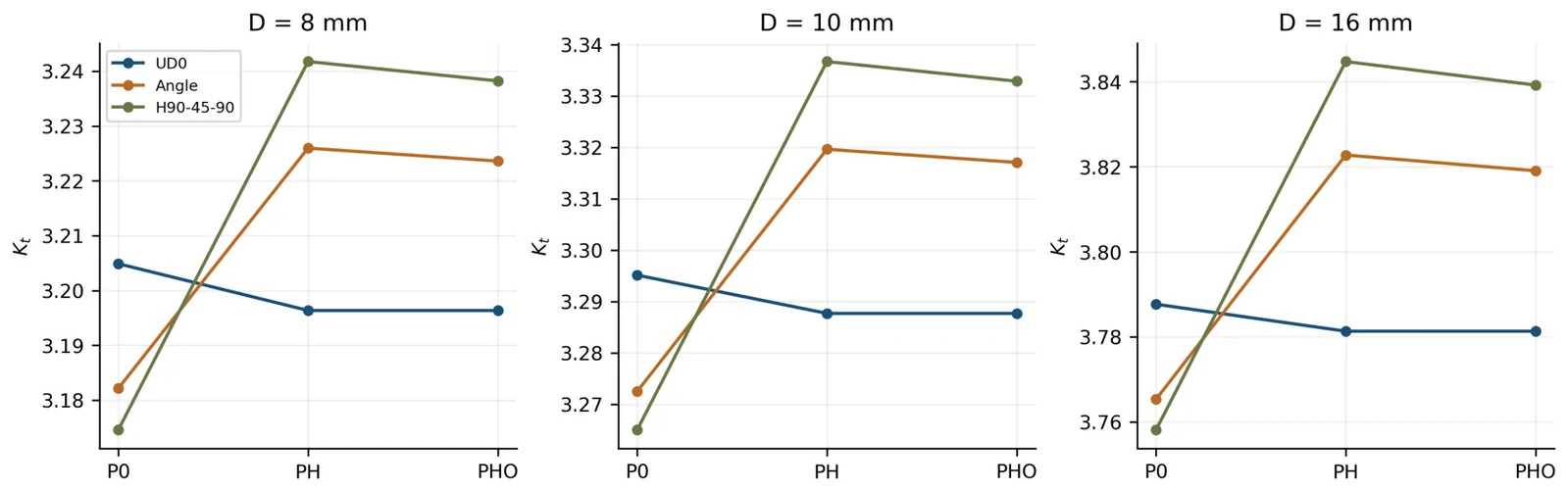
Effects of hole ratio and perimeter representation on stress concentration.

The full six-candidate screen extends this comparison to D/W=0.20 and 0.40. All architectures were included at every geometry, avoiding changes in candidate availability between decision maps. Figure 4 compares the three core architectures across hole ratios and perimeter representations using the common full-integration screen.

**Figure 5:**
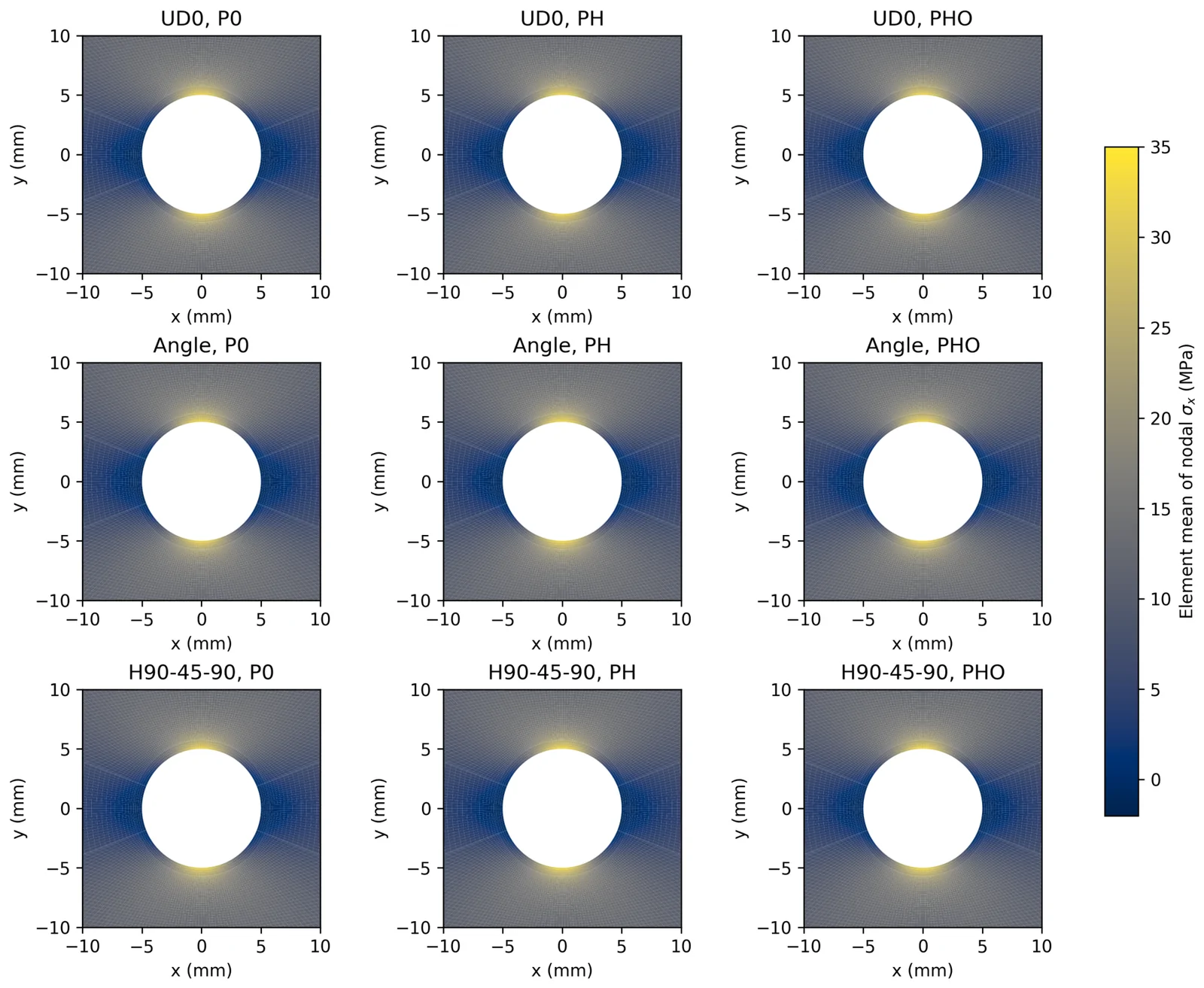
Longitudinal stress fields for the core architectures and perimeter models.

Figure 5 shows the three core architectures’ P0, PH, and PHO longitudinal-stress fields on a common scale in MPa, allowing local redistribution to be compared directly.

### 3.3. Agreement with Published Tensile Curves

The two recoverable experimental curves gave apparent elastic slopes of 2612.9 and 2674.2 MPa. Table 7 compares these with refined PLANE183 predictions. With the unchanged primary Li dataset, the free-end and transversely restrained-end predictions are 2570.5 and 2598.9 MPa. Their slope discrepancies are 0.5–3.9%, while pointwise MAPE is 5.3–7.8%. The distinction matters: agreement in slope does not remove the experimental intercept or establish agreement over the full loading curve.

Using the experimental study’s own elastic constants gives a larger discrepancy: predicted slopes are 13.2–16.0% lower. Refinement, quadratic interpolation, and transverse end restraint do not close this gap. The primary data PLANE183 stiffness changed by less than 0.0005% between the two finest meshes, so discretization does not explain the source-to-source mismatch. A separately digitized unnotched curve from Figure 12a of [10] gives approximately 2275 MPa, close to that study’s 2330 MPa longitudinal modulus but below the apparent slopes of the hole curves. Differences in strain measurement, specimen response, and curve origin therefore require consideration; the available plotted data do not isolate their individual contributions.

**Figure 6:**
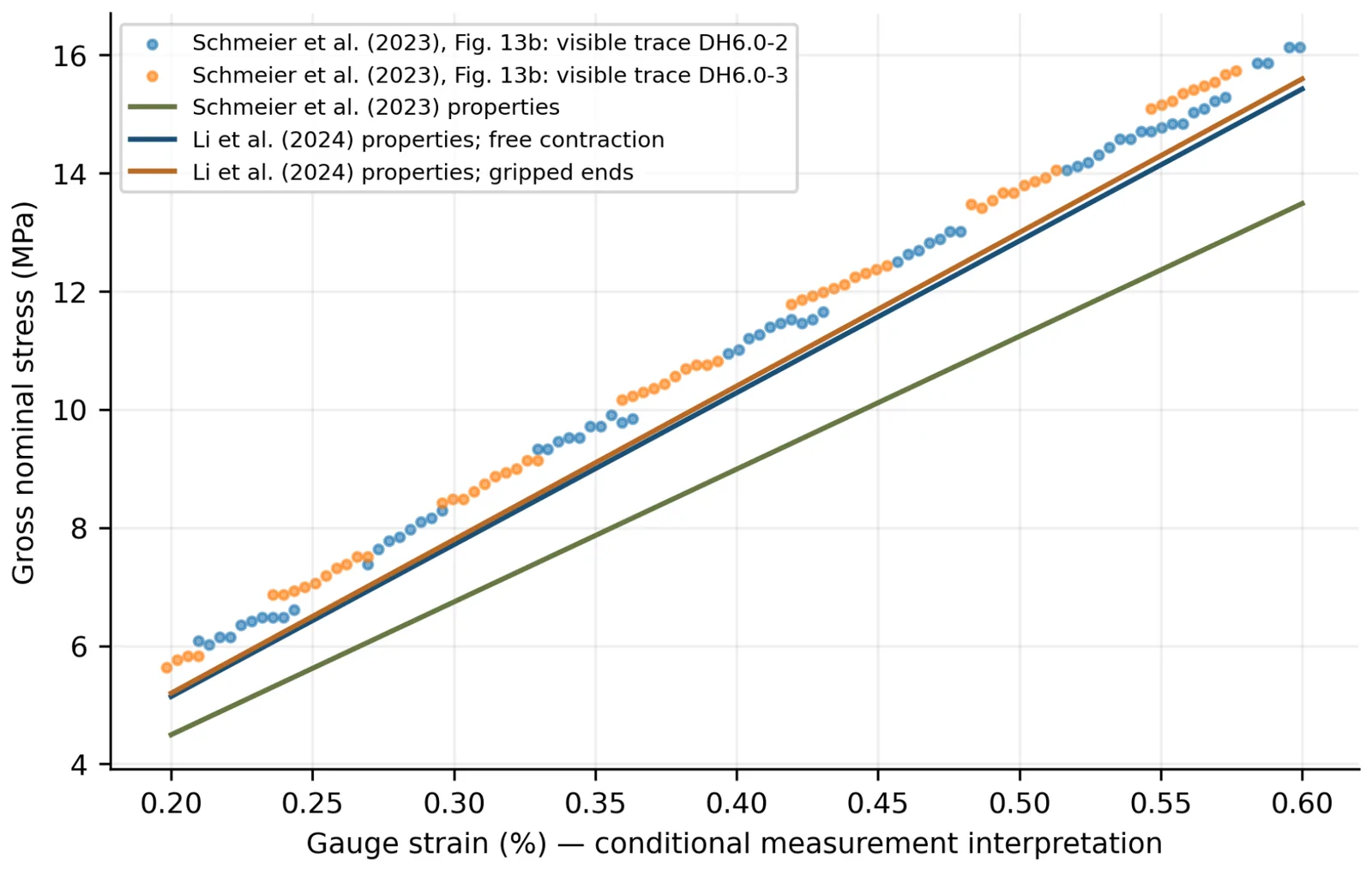
Comparison of predicted and published open-hole elastic responses [1,10].

The smaller errors obtained with Li’s independently selected constants support the scale of the primary model’s global elastic response under the gauge-strain interpretation. This literature-based benchmark is limited to global elastic response; it does not validate local hole-edge stresses, failure loads, or the six-architecture ranking. Two recovered traces and image-reading uncertainty define the evidence basis for this external benchmark. Figure 6 compares the two separately digitized traces over the stated elastic interval with model curves calculated from the independently specified constants. Both property datasets are shown, retaining the less favourable comparison.

### 3.4. Practical Significance, Sensitivity, and Elastic Scope

The primary material has $E_{1}/E_{2}=1.033$, so a large orientation advantage is not expected. Small Young modulus contrast alone does not establish isotropy: $G_{12}$ and Poisson coupling also enter the transformed tensor. With $E_{1}=2669\mathrm{MPa}$ and $\nu_{12}=0.43$, the isotropic relation would give G=933.2MPa rather than the adopted 919 MPa. The predicted sub-percent P0 differences are numerically distinguishable in paired refinement checks, but their practical value depends on larger material and deposition uncertainties. Print-angle and layer-height dependence of elastic properties is supported experimentally [35].

In the retained coarse-mesh property study, the stress-only winner was H90-45-90 in 39 of 100 sets, Angle in 31, and UD0 in 30. At a 3% displacement allowance and stress weight 0.55, the response-constrained selector chose UD0 in 78 sets, Angle in 13, and H90-45-90 in nine. These frequencies characterize the prescribed perturbation design; the 300 solutions retain their common triangular-mesh basis. Together with the perimeter-induced reversal, they show that the small nominal P0 advantage does not support a universal architecture preference.

**Figure 7:**
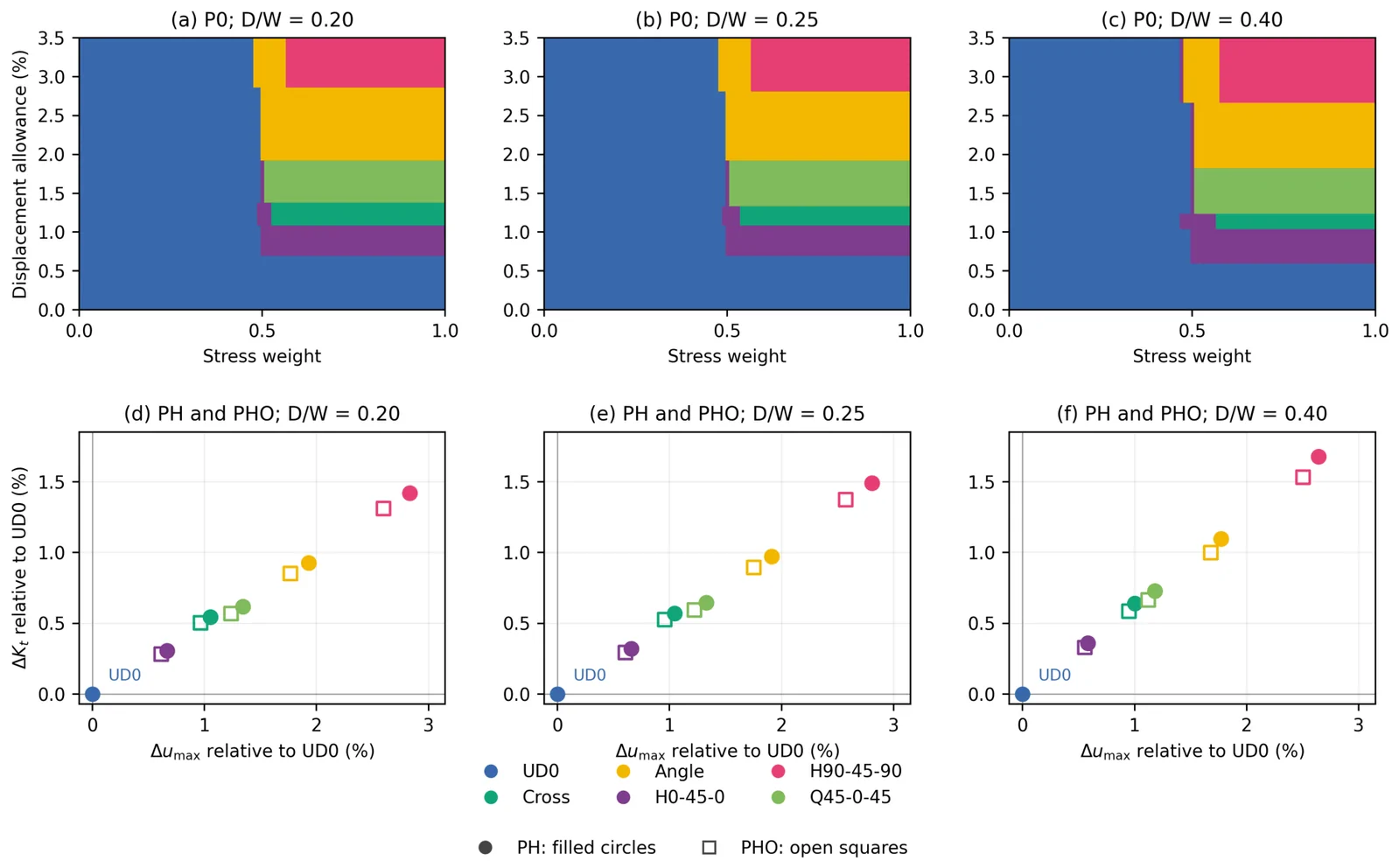
Architecture selection and response differences relative to UD0.

Figure 7 separates the homogeneous-model trade-off from the perimeter-model response. Figure 7a–c show the P0 selection maps, where the preferred architecture depends on stress weight and displacement allowance. In PH and PHO, UD0 has both the lowest $K_{t}$ and the lowest $u_{\max}$ at every tested hole ratio. It therefore dominates the other five candidates and remains selected throughout the investigated weight–allowance domain. Panels (d–f) quantify this result as percentage increases in displacement and stress concentration relative to UD0 at the same geometry and perimeter scenario. Filled circles denote PH and open squares denote PHO. UD0 lies at the origin, and every alternative lies above and to its right. All six panels use the common full-integration screen with a 0.7 mm band. The feasible-set normalization means that a weight has meaning only together with the available candidates, geometry, path representation, and displacement allowance. This is an elastic preference rule, not a strength ranking.

The 10 MPa nominal traction defines the linear-response normalization. Core local longitudinal stresses reach approximately 32– 33 MPa, placing failure assessment outside the present elastic response comparison. For example, the experimental benchmark reports direction-dependent strengths of 57.7 and 23.3 MPa [10], but those values belong to a different print condition and do not define a failure criterion for the primary dataset. Within linear elasticity, multiplying traction multiplies stress and displacement while leaving $K_{t}$ and relative compliance unchanged. Once yielding, bond damage, or cracking begins, this scaling and the elastic ranking need not hold.

Voids and perimeter–infill discontinuities can redirect crack initiation; interlayer fracture can favour a different orientation from the elastic minimum. Fracture reviews, raster-dependent toughness tests, and mixed-mode hole studies support this distinction [27,36,37,38]. The present perfect-bond membrane model resolves elastic stress redistribution, while critical load, crack path, and delamination require a failure-capable representation. Accordingly, a production decision based on a small elastic difference should incorporate the actual deposition path and print-specific tensile, shear, and fracture characterization.

The complete architecture screen, mesh-refinement and shell comparisons, search-radius and reaction checks, digitized elastic curves, and response-constrained decision maps are provided in the Supplementary Materials.

## 4. Conclusions

For homogeneous open-hole PLA models at D/W=0.25, the finest quadrilateral comparison gives a 0.90% stress-concentration reduction for [90/45/90] relative to [0/0/0], accompanied by increased displacement. Tangential hole and outer perimeter bands reverse that stress ordering, with $K_{t}=3.2660$ for [0/0/0] and 3.3109 for [90/45/90]. Local deposition direction is therefore essential to interpreting the small advantage predicted by a uniform equivalent laminate.

Paired mesh refinement, an independent block topology, and matched layered-shell comparisons support the numerical ordering within the stated membrane models. The unchanged primary elastic constants reproduce the slopes of two published open-hole traces within 0.5–3.9%, with pointwise errors of 5.3–7.8%. The larger 13.2–16.0% slope discrepancy obtained using the experimental study’s own constants demonstrates the remaining dependence on constitutive data and strain interpretation. These comparisons support global elastic response for the specified benchmark. The finite-element comparisons resolve the architecture ranking within the stated membrane models. Architecture selection should state the perimeter path, material data, and displacement allowance alongside its predicted stress benefit; failure resistance requires the corresponding failure characterization.

## Figures and Tables

**Table 1:** In-plane elastic constants used in the plane-stress model.

$E_{1}$ (MPa)	$E_{2}$ (MPa)	$G_{12}$ (MPa)	$\nu_{12}$
2669	2583	919	0.43

**Table 2:** Thickness-normalized extensional stiffnesses for the three-ply candidates (MPa).

Sequence	$Q_{11}^{\mathrm{eq}}$	$Q_{22}^{\mathrm{eq}}$	$Q_{12}^{\mathrm{eq}}$	$Q_{66}^{\mathrm{eq}}$	$Q_{16}^{\mathrm{eq}}$	$Q_{26}^{\mathrm{eq}}$
[0/0/0]	3250.7	3145.9	1352.8	919.0	0.0	0.0
[0/90/0]	3215.8	3180.9	1352.8	919.0	0.0	0.0
[45/−45/45]	3194.5	3194.5	1356.5	922.8	8.7	8.7
[0/45/0]	3232.0	3162.1	1354.0	920.3	8.7	8.7
[90/45/90]	3162.1	3232.0	1354.0	920.3	8.7	8.7
[45/0/45]	3213.3	3178.3	1355.3	921.5	17.5	17.5

**Table 3:** Architecture and perimeter codes. Angles are in degrees from the loading direction.

Code	Definition
UD0	[0/0/0]
Cross	[0/90/0]
Angle	[45/−45/45]
H0-45-0	[0/45/0]
H90-45-90	[90/45/90]
Q45-0-45	[45/0/45]
P0	Uniform equivalent-laminate stiffness throughout
PH	Tangential hole band; equivalent-laminate interior
PHO	Hole band and edge-parallel outer band; laminate interior

**Table 4:** Matched P0 plane and shell results at D/W=0.25 and $n_{\theta }=512$.

Architecture	$K_{t}^{\mathrm{plane}}$	$K_{t}^{\mathrm{shell}}$	$\delta_{K_{t}}$ (%)	$\delta_{u}$ (%)
UD0	3.27798	3.27703	−0.02905	−0.001091
Angle	3.25566	3.25477	−0.02717	−0.001088
H90-45-90	3.24840	3.24751	−0.02718	−0.001086

**Table 5:** Baseline six-candidate results, full-integration PLANE182, $n_{\theta }=512$, band width 0.7 mm.

Architecture	P0		PHO	
	$K_{t}$	$u_{\max}$ **(mm)**	$K_{t}$	$u_{\max}$ **(mm)**
UD0	3.29514	0.399665	3.28770	0.399751
Cross	3.28391	0.403893	3.30503	0.403580
Angle	3.27250	0.407394	3.31708	0.406751
H0-45-0	3.28761	0.402319	3.29740	0.402172
H90-45-90	3.26501	0.411019	3.33289	0.410042
Q45-0-45	3.28010	0.405029	3.30728	0.404642

**Table 6:** Finest core comparison, enhanced-strain PLANE182, $n_{\theta }=1024$, D/W=0.25, band width 0.7 mm.

Architecture	P0		PHO	
	$K_{t}$	$u_{\max}$ **(mm)**	$K_{t}$	$u_{\max}$ **(mm)**
UD0	3.27315	0.399652	3.26596	0.399732
Angle	3.25090	0.407381	3.29517	0.406706
H90-45-90	3.24372	0.411006	3.31089	0.410020

**Table 7:** Experimental elastic comparison. Each error pair refers to the two digitized traces, in the same order. Signed slope errors are negative because the predicted slope is lower.

Dataset	Transverse Ends	$E_{\mathrm{app},\mathrm{FE}}$ (MPa)	Slope Error (%)	MAPE (%)
Li	Free	2570.5	−1.62,−3.88	6.37,7.81
Li	Restrained	2598.9	−0.53,−2.81	5.33,6.79
Schmeier	Free	2247.2	−13.99,−15.97	18.15,19.40
Schmeier	Restrained	2267.2	−13.23,−15.22	17.42,18.69

## Data Availability

The original contributions presented in this study are included in the article/Supplementary Material. Further inquiries can be directed to the corresponding author.

## Supplementary Materials

The following supporting information can be downloaded at https://www.mdpi.com/article/10.3390/polym18182301/s1. Supporting data include the complete six-architecture screen, core mesh-refinement and shell comparisons, search-radius and reaction checks, digitized elastic curves and error measures, and the response-constrained decision maps.
